# Depression and post-traumatic stress disorder among Haitian immigrant students: implications for access to mental health services and educational programming

**DOI:** 10.1186/1471-2458-9-482

**Published:** 2009-12-22

**Authors:** Mary C Smith Fawzi, Theresa S Betancourt, Lilly Marcelin, Michelle Klopner, Kerim Munir, Anna C Muriel, Catherine Oswald, Joia S Mukherjee

**Affiliations:** 1 Program in Infectious Disease and Social Change, Department of Global Health and Social Medicine, Harvard Medical School, Boston, MA, USA; 2 Partners In Health, Boston, MA, USA; 3 Department of Global Health and Population, Harvard School of Public Health, Boston, MA, USA; 4 Haitian Mental Health Clinic, Cambridge Hospital, Cambridge, MA (former affiliation), USA; 5 Children's Hospital Boston, Boston, MA, USA; 6 Department of Psychiatry, Massachusetts General Hospital, Boston, MA, USA

## Abstract

**Background:**

Previous studies of Haitian immigrant and refugee youth have emphasized "externalizing" behaviors, such as substance use, high risk sexual behavior, and delinquency, with very little information available on "internalizing" symptoms, such as depression and anxiety. Analyzing stressors and "internalizing" symptoms offers a more balanced picture of the type of social and mental health services that may be needed for this population. The present study aims to: 1) estimate the prevalence of depression and post-traumatic stress disorder (PTSD) among Haitian immigrant students; and 2) examine factors associated with depression and PTSD to identify potential areas of intervention that may enhance psychosocial health outcomes among immigrant youth from Haiti in the U.S.

**Methods:**

A stratified random sample of Haitian immigrant students enrolled in Boston public high schools was selected for participation; 84% agreed to be interviewed with a standardized questionnaire. Diagnosis of depression and PTSD was ascertained using the best estimate diagnosis method.

**Results:**

The prevalence estimates of depression and PTSD were 14.0% and 11.6%; 7.9% suffered from comorbid PTSD and depression. Multivariate logistic regression demonstrated factors most strongly associated with depression (history of father's death, self-report of schoolwork not going well, not spending time with friends) and PTSD (concern for physical safety, having many arguments with parents, history of physical abuse, and lack of safety of neighborhood).

**Conclusions:**

A significant level of depression and PTSD was observed. Stressors subsequent to immigration, such as living in an unsafe neighborhood and concern for physical safety, were associated with an increased risk of PTSD and should be considered when developing programs to assist this population. Reducing exposure to these stressors and enhancing access to social support and appropriate school-based and mental health services may improve educational attainment and psychosocial health outcomes among Haitian immigrant youth.

## Background

Refugee and immigrant children have a broad range of needs, including adequate housing, healthcare, food, education, and basic security [1]. As part of their healthcare needs, refugee and immigrant children also require adequate access to mental health services and support programming that can serve as primary and/or secondary prevention of poor psychosocial health outcomes. These outcomes can consist of depression, anxiety, post-traumatic stress disorder, conduct problems, difficulties relating to adults and/or peers, attention problems, difficulty concentrating, and poor school performance [2-10].

Despite the fact that there is a critical need for mental health services and related programming for refugee and immigrant youth, there are barriers to accessing care that often include language as well as finances. For example, immigrant children in Canada have a lower likelihood of mental health/social services utilization compared to non-immigrant children (relative odds of 0.39, p = 0.02) [11]. In addition to the underutilization of mental health services, immigrant and refugee youth have also demonstrated a greater need for mental health services compared to the host population in the Netherlands [12], the United Kingdom [13], and Switzerland [14].

While the broader body of knowledge on the mental health of refugee and immigrant youth focuses on a range of psychosocial health outcomes [2-6], studies of Haitian immigrant and refugee youth have emphasized "externalizing" behaviors, such as substance use [15], high risk sexual behavior [16], delinquency [17], and "deviant behavior" [18,19], with very little information available on "internalizing" symptoms, such as depression and anxiety, among Haitian youth. A focus on externalizing problems presents a skewed view of Haitian youth and does not address the root causes of such behaviors. Analyzing stressors and "internalizing" symptoms, such as depression and anxiety, offers a more balanced picture of the type of social and mental health services that may be needed. For example, one study indicated that the mean "internalizing" score on the Youth Self-Report was higher for Haitian youths compared with Latino and African American youths 12-18 years old attending Boston youth groups (p < 0.004) [20]. Douyon et al. [21] observed a 27.6% prevalence of PTSD among Haitian immigrant youth in Miami/Dade County, however, the researchers were primarily interested in the association of trauma exposure, PTSD, and involvement in "drug-involved social groups." A study in the greater Miami area examined the relationship between substance use and suicidal ideation; the authors found a high level of suicide attempts (11.4%) in their Haitian sub-sample of boys attending middle-school [22].

In addition, Haitian immigrant youth in the U.S. have experienced significant levels of poverty. Among a study population of Haitian immigrants in Florida, 28% reported a family income of less than $10,000 annually [23]. Family characteristics from another study with Haitian immigrants indicated that 26% earned less than $20,000 per year and 22% were unemployed; only 50% were working full-time at the time of the survey [24]. This level of poverty also has significant implications for the mental health of Haitian immigrant youth.

Given the high level of suicidality documented previously [22], the elevated rates of poverty [23,24], and the relative lack of knowledge of "internalizing" symptoms among Haitian immigrant and refugee youth, the primary aim of this study is to estimate the prevalence of depression and post-traumatic stress disorder (PTSD) among Haitian immigrant students enrolled in public schools in an urban center in the U.S. By selecting students enrolled in school, this study offers the opportunity to inform potential interventions that can assist Haitian immigrant youth within public school systems. Through examination of factors associated with depression and PTSD in this population, a secondary aim is to identify potential areas of intervention that may enhance psychosocial health outcomes among immigrant youth from Haiti and similar settings of poverty, instability, and violence. Due to these broad systemic problems, as well as HIV/AIDS, the number of vulnerable children who have faced significant life trauma is growing. Studies that examine factors associated with poor psychosocial health outcomes among children and youth at high risk offer data that can inform programs aimed at reducing suffering, improving quality of life, and increasing chances of a promising transition to adulthood.

## Methods

### Study population

Immigrant youth from Haiti were recruited from bilingual programs at Boston public high schools (grades 10-12). Bilingual teachers completed the Teacher's Report Form (TRF) [25] to provide initial screening information. The TRF is analogous to the Child Behavior Checklist (CBCL) developed by Achenbach that inquires about a broad range of internalizing and externalizing psychological symptoms in children; however, the responses for the TRF are based on teacher's report rather than child self-report [25]. Based on an initial sample of 315 students, we randomly selected a sub-sample of 100 students who had scores on or above the TRF borderline clinical cut-off and 100 students who had scores below this cut-off score. This cut-off score was derived from Achenbach's work validating the CBCL and related scales (e.g. TRF) based on empirical data collected from clinical and community-based samples. The borderline clinical cut-off score range was determined based on this validation work. Youth who achieved a cut-off score at or above the lower borderline cut-off score were considered as having a level of symptoms comparable with those receiving clinical care or those on the threshold of needing this type of care [25].

The rationale for the stratified sampling strategy was based on an additional objective beyond the scope of the current paper to perform validation analyses on the psychiatric symptom scales used in the study. For the 200 students selected, an interviewer approached each student to invite him/her to participate in the study. Eighty-four percent agreed to participate (n = 168). The main reason for not participating was the interviewer's inability to locate the prospective participant by telephone. In some cases, the students did not return telephone calls and in other cases the telephone number had been changed without any available information on the new number. The study enrollment period was from July 1998 to November 2000. Students were interviewed if informed consent was obtained. For participants under the age of 18 years, informed consent was obtained from a parent or legal guardian and assent was obtained from the students. Informed consent and procedures for the study were approved by the Institutional Review Board at the Harvard School of Public Health. In addition to following a standard protocol regarding human subjects protection, we actively referred students for healthcare, mental health, and educational services when appropriate. Among those who participated in the study, 39 were referred for services. Participants were generally receptive to receive assistance, aside from several students who demonstrated a significant degree of externalizing behaviors.

### Measures and assessment

A standardized interview was performed by a trained interviewer, fluent in Haitian Creole and English, with all students enrolled in the study. All interviews were conducted in Haitian Creole, the study participants' first language, and were approximately one hour in duration. The questionnaire was translated by a professional translator with the exception of sections that included psychiatric symptoms; these sections were translated by a Haitian clinical psychologist to ensure conceptual equivalence of the items that involved psychological content. The interview included the following sections: sociodemographic and economic information; language skills (Haitian Creole and English); self-reported academic performance; social functioning; social support; negative life events, including traumatic events; risk of physical injury (to self or others); HIV risk behavior; substance use history; and psychiatric symptoms. Scales in the questionnaire that had psychiatric symptoms included the Youth Self-Report (YSR) [25]; the Children's Depression Inventory (CDI) [26]; and the Child Post-Traumatic Stress Reaction Index (CPTSRI) [27]. Since the CPTSRI was developed based on DSM-III-R criteria, seven more items were added to accommodate DSM-IV criteria for PTSD [28]. In addition, risk behavior was assessed using the Youth Risk Behavior Surveillance System (YRBSS) developed by the Centers for Disease Control and Prevention [29].

Based on the information collected in the interview, a best estimate diagnosis (BED) for depression, PTSD, and other mental disorders was ascertained by board-certified child psychiatrists [30-32]. Diagnoses were based on current symptomatology according to self-report in the standardized interview. The structured protocol for determining the BED included an initial assessment by a child psychiatrist with international experience (first rater). If no diagnoses were indicated by this rater then the BED was determined by the first rater. If there was at least one positive diagnosis, a second rating was performed by a different child psychiatrist (second rater). If substantial agreement was present between these two ratings, then the initial assessment (first rater) was used as the BED. Substantial agreement was defined as agreement for PTSD, depression, and attention deficit hyperactivity disorder (ADHD) diagnoses. If substantial agreement was not present, then a third rater (another child psychiatrist) reviewed the initial questionnaire and the other two raters' assessments to provide the BED. Twenty-one participants required the assessment of this third rater. The third rater's level of confidence in each diagnosis was indicated by the following categories: possible, probable, and definite [32]. Clinicians based their assessment of depression on self-report symptoms from the CDI and the relevant items from the YSR (e.g. withdrawal and anxious/depressed sub-scales); symptoms from the CPTSRI were used for the BED of PTSD.

### Statistical analysis

General descriptive statistics were calculated for sociodemographic and economic characteristics. Frequencies were assessed for symptoms and best estimate diagnoses of depression and PTSD. For bivariate analyses of depression, PTSD and related risk factors, odds ratios and corresponding 95% confidence intervals were estimated; p-values were based on chi-square statistics or Fisher's exact test when expected cell counts were less than five. Multiple logistic regression was performed to identify risk factors that demonstrated the strongest prediction of depression and PTSD in this population. Since the study was exploratory in nature and inquired about a broad range of relevant factors, the forward selection technique was utilized. Factors considered for inclusion in the analysis were sociodemographic characteristics (e.g. age, gender, employment, financial situation, etc.); school performance and attendance; quality of relationships with family members and friends, including social support; death of a parent; family history of substance abuse or suicidality; past trauma events and current level of safety of neighborhood. Data were analyzed using SAS Version 8.2 for Windows [33].

## Results

Among 200 students who were selected to participate in the study, 168 agreed to be interviewed. The students were 15 to 24 years of age (mean = 18.8 years) and enrolled in grades 10 through 12, with a majority in the 12^th ^grade (69%). Nearly 54% of the sample was female and the primary language spoken at home was Haitian Creole (98%). On average, they had been living in the U.S. for four years (range 1-11 years). Over 70% were living with their mother at the time of the study; however, less than 50% lived with their father. Over 59% mentioned that their families had experienced financial difficulties (see Table 1).

**Table 1:** Sociodemographic characteristics of 168 Haitian immigrant students attending public high schools in the Boston area

Characteristics	n (%)*
*Categorical variables*	
Gender	
Female	90 (53.6%)
Male	78 (46.4%)
Grade	
10^th^	16 (9.6%)
11^th^	36 (21.6%)
12^th^	115 (68.9%)
Primary language spoken at home	
Haitian Creole	164 (97.6%)
English	4 (2.4%)
Ability to speak English	
Very well	123 (74.1%)
Some/very little/not at all	43 (25.9%)
Ability to read English	
Very well	132 (78.6%)
Some/very little	36 (21.4%)
	
Currently living with:	
Mother	116 (70.7%)
Father	69 (45.4%)
Family owns/rents home	
Owns home	16 (10.7%)
Rents home	133 (89.3%)
Safety of neighborhood	
Very/somewhat unsafe	36 (22.5%)
Fairly/very safe	124 (77.5%)
Family having financial difficulties	
Yes	97 (59.1%)
No	67 (40.9%)
Youth currently employed	
Full-time (40-50 hours per week)	3 (1.8%)
Part-time (20-39 hours per week)	28 (16.7%)
Part-time (<20 hours per week)	92 (55.1%)
Not employed	44 (26.4%)
	
*Continuous variables*	Mean (SD) (range)
Age (years) (n = 167)	18.8 (1.58) (15-24)
Number of years living in U.S. (n = 168)	4.0 (1.67) (1-11)
Number of people living in household (n = 164)	5.0 (1.56) (2-9)
Number of children living in household (n = 166)	1.4 (1.11) (0-5)
Number of rooms in household (n = 164)	2.6 (0.75) (1-7)
Household density (# people/#rooms) (n = 159)	2.0 (0.62) (0.6-4.5)
	

*Percent may not reflect denominator of 168 for some characteristics due to missing data.

In terms of traumatic or highly stressful life events, nearly 5% of the students had at least one parent die. Approximately 10% indicated a history of physical or sexual abuse. Overall, 16% reported having experienced at least one traumatic event. Six percent indicated they felt concerned for their physical safety (at the time of the study) and nearly 4% said that they currently did not feel safe at home. Approximately 4% of the students had a history of suicidal thoughts and over 2% had previously attempted suicide. Over 4% reported that they had a friend who attempted suicide in the past. See Table 2 for a description of the traumatic and/or stressful life events experienced by the students who participated in the study.

**Table 2:** Lifetime and one-year prevalence of traumatic and/or stressful life events experienced by Haitian immigrant students

Event	Prevalence, n (%)	
	Lifetime (n = 166)*	Past Year (n = 166)*
You changed schools	99 (59.6%)	7 (4.2%)
Your parents were very strict or harsh with you	92 (55.4%)	78 (47.5%)
A new person moved into your home	47 (28.3%)	9 (5.4%)
You broke up with a girlfriend or boyfriend	44 (26.8%)	10 (6.1%)
Had many arguments with your mother or father	43 (26.1%)	26 (15.6%)
A close friend moved away	36 (21.7%)	7 (4.2%)
You moved out of your house	28 (16.9%)	13 (7.8%)
A family member moved out of your house	25 (15.1%)	2 (1.2%)
You repeated a grade	23 (13.9%)	5 (3.0%)
You failed a class	22 (13.3%)	8 (4.8%)
Your parents were having many arguments	17 (11.5%)	7 (4.7%)
You had difficulties in school	17 (10.2%)	12 (7.2%)
		
Another family member died	12 (7.3%)	2 (1.2%)
You were slapped, hit or beaten by another person	12 (7.3%)	8 (4.8%)
Had many arguments with other family members	12 (7.2%)	4 (2.4%)
Had many arguments with your girlfriend or boyfriend	12 (7.2%)	3 (1.8%)
You were teased or "picked on" by classmates	11 (6.6%)	2 (1.2%)
Your parents separated	10 (6.5%)	4 (2.6%)
You lost a job	10 (6.1%)	3 (1.8%)
A close friend died	6 (3.6%)	3 (1.8%)
Your parents got divorced	5 (3.2%)	2 (1.9%)
Your father died	4 (2.4%)	4 (2.4%)
You had a serious illness or injury	4 (2.4%)	1 (0.6%)
You were threatened by another person	4 (2.4%)	2 (1.2%)
You were physically abused or assaulted by another person	4 (2.4%)	1 (0.6%)
		
One of your parents remarried	2 (1.9%)	51 (32.4%)
You were suspended from school	3 (1.8%)	4 (2.4%)
A parent had a serious illness or injury	3 (1.8%)	3 (1.8%)
You were mugged	3 (1.8%)	1 (0.6%)
Had many arguments with friends	3 (1.8%)	4 (2.4%)
You ran away from home	3 (1.8%)	2 (1.2%)
Your mother died	2 (1.2%)	2 (1.2%)
Your friends ignored you or left you out of activities	2 (1.2%)	2 (1.2%)
You were attacked with a knife or stabbed	2 (1.2%)	2 (1.2%)
You were shot at or shot	2 (1.2%)	2 (1.2%)
Had physical fights	1 (0.6%)	0 (0.0%)
Another family member had a serious illness or injury	1 (0.6%)	1 (0.6%)
A close friend had a serious illness or injury	1 (0.6%)	1 (0.6%)

*Percent may not reflect denominator of 166 for some items due to missing data.

Table 3 reports on depression, PTSD, and frequencies for specific symptoms. Overall, 14% of the students demonstrated symptoms suggestive of depression according to the BED. Approximately 15% said they were sad many times or all the time and over 41% said that they felt somewhat or very lonely. The most common complaints were "I have to push myself all the time/many times to do my school work" (39%) and "my schoolwork is not as good as before/I do very badly in subjects I used to be good in" (22%). For PTSD, nearly 12% of the students had symptoms that suggested diagnosis according to the best estimate method used. For specific symptoms, 48% indicated that the "event was something that would upset or bother most children a lot." Over 46% responded "no" to the question "Is it as easy to pay attention as before the event?" which is a post-trauma symptom that can have implications for school performance. Results from Table 3 suggest that youth experienced the complete range of symptoms of intrusion, avoidance, and arousal that characterize PTSD. Nearly 8% of this population of Haitian youth demonstrated comorbid PTSD and depression. Despite this significant prevalence of symptoms of depression and PTSD, only 1.8% of the study population reported having seen a mental health professional.

**Table 3:** Symptoms and diagnoses of depression and PTSD among Haitian immigrant students

Symptom	n (%)
*Depression* (Children's Depression Inventory n = 165)*	
	
I have to push myself all the time/many times to do my schoolwork	65 (39.4%)
My schoolwork is not as good as before/I do very badly in subjects I used to be good in	36 (21.8%)
I feel alone many times/all the time	33 (20%)
I never have fun at school or fun only once in a while	26 (15.8%)
I have some friends but wish I had more/I do not have any friends	26 (15.8%)
Things bother me all the time/many times	25 (15.2%)
Sad many times or all the time	25 (15.2%)
I feel like crying many days/every day	19 (11.6%)
	
(Youth Self-Report n = 168)** (selected items reporting somewhat or very true)	
	
I feel that I have to be perfect	101 (60.5%)
Lonely	70 (41.7%)
I am nervous or tense	24 (14.5%)
I am too fearful or anxious	20 (12.0%)
	
**Depression (diagnosis; n = 164)**	**23 (14.0%)**
	
Post-traumatic stress disorder (n = 168)**	*(% reported 'a little' or more unless otherwise specified)*
Event something that would upset or bother most children a lot	80 (47.6%)
Is it as easy to pay attention as before the event? (% reporting 'no')	78 (46.4%)
Do you feel more alone inside, or more alone with your feelings?	69 (41.1%)
Do thoughts of event come back to you even when you don't want them to?	65 (38.7%)
When something reminds you, or makes you think about the event, do you get tense or upset?	64 (38.1%)
Do you get scared, afraid or upset when you think about event?	63 (37.5%)
Do things sometimes make you think it might happen again?	54 (32.1%)
Do you feel irritable or have outbursts of anger?	53 (31.5%)
Do you go over in your mind what happened?	51 (30.4%)
	
Do you have good or bad dreams of event or other bad dreams?	50 (29.8%)
Do you feel as good about things you liked to do before the event? (% reporting 'no')	50 (29.8%)
Have you felt so scared, upset, or sad that you couldn't even talk or cry?	49 (29.2%)
Do you want to stay away from things that make you remember what happened to you?	46 (27.4%)
Do you startle more easily or feel more jumpy or nervous than before the event?	43 (25.6%)
Do you avoid thoughts or feelings associated with the traumatic or hurtful events?	40 (23.8%)
Do you feel on guard or extremely aware of what is going on around you?	38 (22.6%)
Do you have recurrent dreams of hurtful or traumatic events?	36 (21.4%)
Do you feel so scared, upset, or sad that you don't really want to know how you feel?	34 (20.2%)
Do you ever feel as if the hurtful or traumatic events were happening all over again?	28 (16.7%)
Do you have more stomach aches, headaches, or other sick feelings since the event than before?	28 (16.7%)
Do thoughts or feelings about what happened get in the way of remembering things?	21 (12.5%)
	
**PTSD (diagnosis; n = 164)**	**19 (11.6%)**
	
**Depression and PTSD (comorbidity; n = 164)**	**13 (7.9%)**

*Percent may not reflect denominator of 165 for some characteristics due to missing data.

**Percent may not reflect denominator of 168 for some characteristics due to missing data.

Factors associated with BED of depression and PTSD were initially examined through bivariate analyses. The factors that demonstrated the strongest associations with depression were history of physical or sexual abuse (OR = 9.0; 95% confidence interval (CI): 2.8, 28.7), self-report of schoolwork not going well (OR = 11.9; 95% CI: 4.2, 33.7), and low GPA (OR = 11.0; 95% CI: 2.5, 49.2). The ability to go to their mother if they needed help with a serious problem reduced the risk of depression by 74% (OR = 0.26; 95% CI: 0.10, 0.69). In terms of PTSD, the strongest factors associated were history of physical abuse (OR = 13.7; 95% CI: 4.0, 46.8), history of physical or sexual abuse (OR = 11.9; 95% CI: 3.6, 39.5), having many arguments with parents (OR = 10.9; 95% CI: 3.3, 35.8), feeling rejected by someone (OR = 9.4; 95% CI: 2.9, 29.9), and present concern for physical safety (OR = 8.6; 95% CI: 2.0, 35.8). The ability to go to their mother or father if they needed help with a serious problem reduced the risk of PTSD by 68% (OR = 0.32; 95% CI: 0.11, 0.97) in bivariate analyses.

Results from multivariate analyses using multiple logistic regression and the forward selection technique are presented in Table 4. Factors that remained in the final model for depression included self-report of schoolwork not going well (OR = 19.9; 95% CI: 4.9, 81.3), history of father's death (OR = 14.6; 95% CI: 1.5, 142.3), not spending time with friends (OR = 5.1; 95% CI: 1.3, 19.7), and having many arguments with parents (OR = 4.1; 95% CI: 1.2, 13.8). For PTSD, factors in the final model were: concern for physical safety (OR = 38.9; 95% CI: 3.8, 393.3), having many arguments with parents (OR = 26.8; 95% CI: 4.4, 162.6), history of physical abuse (OR = 13.6; 95% CI: 2.2, 85.1), and lack of safety of neighborhood (OR = 11.9; 95% CI: 2.2, 65.8).

**Table 4:** Multivariate analysis of factors associated with depression and PTSD among Haitian immigrant students

Model	Odds ratio (95%CI)
*Depression* (n= 143)	
Self-report of how schoolwork is going (not well vs. well)	19.9 (4.9, 81.3)
Hanging out with friends (none vs. some)	5.1 (1.3, 19.7)
Having many arguments with mother or father (yes vs. no)	4.1 (1.2, 13.8)
Father died (yes vs. no)	14.6 (1.5, 142.3)
	
*Post-traumatic stress disorder* (n = 137)	
Safety of neighborhood (unsafe vs. safe)	11.9 (2.2, 65.8)
Having many arguments with mother or father (yes vs. no)	26.8 (4.4, 162.6)
History of physical abuse (yes vs. no)	13.6 (2.2, 85.1)
Concerned for physical safety (yes vs. no)	38.9 (3.8, 393.3)

## Discussion

In the present study, we observed a prevalence of depression and PTSD of 14.0% and 11.6%, respectively; 7.9% of students suffered from comorbid PTSD and depression. Existing literature from refugee, immigrant and displaced youth indicates a wide range of prevalence estimates for depression and PTSD. Prevalence estimates for depression range from 8.4% in adolescent refugees resettled in Quebec [34] to 68.7% among Cambodian youth at the Site II refugee camp in Thailand [35]. For PTSD, prevalence rates were from 9% among Cambodian adolescents living in a west coast city in the U.S. [6] to 93.8% among youth displaced by war living in collective centers in Bosnia [36]. In a review article of the literature on refugee mental health by Fazel et al. [2], a summary prevalence of 11% for PTSD among children and youth was estimated, similar to the finding from the present study. While Haitian immigrants in the U.S. are not typically considered as "refugees" to a large extent, given the context of a high degree of insecurity and political violence in Haiti and the extreme level of poverty it is not surprising that the Haitian immigrant students in this sample have a similar level of PTSD compared to other groups of refugee children and youth. For many Haitian immigrants in the U.S., the differentiation between being an economic immigrant versus a refugee fleeing political strife is often not relevant, given that in many cases those who fear political persecution often also suffer from severe economic deprivation [37,38].

Risk factors for depression among Haitian immigrant youth in the present study for the final multivariate model included self-report of schoolwork not going well, not having opportunities to spend time with friends, having many arguments with parents, and father's death. Considering the father's death as a highly traumatic event, this finding is corroborated by existing literature in refugee and immigrant youth with respect to an association between trauma and depression. Traumatic events have been associated with depression among Cambodian adolescents in Oregon [39], refugee children in London [40], Latin American immigrant children [41] and Somalian adolescent refugees [8] in the U.S. Similar to findings in the present study, factors predicting anxiety and depression among Vietnamese Amerasian youth included a history of missing school or lack of school attendance, negative or indifferent feeling toward father, hostile relationship with step/foster father, and not always living with mother [42]. Fazel and Stein [43] also indicate that declining school performance can serve as a symptom of depression in refugee youth. Alternatively, academic difficulties due to language barriers or other problems related to adjustment may affect a young person's sense of self-efficacy, also potentially contributing to depression [44]. Finally, similar to findings from the present study, a higher level of social support was associated with a lower level of depressive symptoms among unaccompanied minors from Southeast Asia [45] as well as Namibian adolescent refugees [46].

For PTSD, factors retained in the final multivariate model for the present study included living in an unsafe neighborhood, having many arguments with parents, history of physical abuse, and current concern for physical safety. Consistent with results from the present study, many prior studies among refugee youth have demonstrated an association between traumatic events and PTSD [4,47-49]. While these studies examined the overall association between trauma or violence exposure and PTSD, very few studies have examined the level of violence exposure in the immigrants' home countries versus the United States or other resettlement country. Among Cambodian refugee adolescents, 25% reported that they had survived violence directed at them outside of the U.S.; however, 29% also reported that they survived violence directed at them in the U.S. [6]. The magnitude of the association between the level of trauma and number of PTSD symptoms was greater for violence experienced in the U.S. (r = 0.362, p < 0.01) compared with the violence experienced overseas (r = 0.192; p > 0.05); these results persisted in the multivariate regression analysis that controlled for other variables [6]. Post-traumatic stress reactions were also associated with adolescents perceiving their mother's parenting style as "rejecting" (r = 0.30) [50], similar to results from the present study.

Findings from this study have implications for identifying much-needed services for refugee and immigrant youth. Given the fact that school performance is important and highly associated with depression, development of English language skills may help to address this issue. Language development and second language learning has been put forth as essential for immigrant youth enrolled in schools in Canada; students often feel less isolated and more connected to their new environment [51]. It is important to note however, that symptoms of PTSD may have an impact on developing second language skills [52]. A similar impact of depression on language skills may also be observed given the effect of depression on cognition [28]. A lower level of English language skills was associated with a higher level of major depression symptomatology (β = -0.234, p < 0.05) among Cambodian refugee adolescents in the U.S. [52]. However, efforts to enhance English language skills should not be promoted in exclusion of much-needed bilingual programs in the schools.

Social support or the demonstrated lack thereof (father's death, arguments with parents) was strongly associated with depression and PTSD in this population of Haitian immigrant youth. In addition, univariate analyses demonstrated a strong protective effect of parental support. A number of prior studies have documented the role of social support as a protective factor [53-56]. In particular, having a confidante that provides emotional support has been shown to have a buffering effect on depression among women [53]. For young children the primary confidante may be a parent; however, for older children and youth this may shift to support from a peer. Harding and Looney [54] observed that Vietnamese children in a refugee camp demonstrated a greater capacity to cope if they were supported by their families. Separation from families indicated a greater vulnerability to stress [57]. These findings are parallel to the earlier observations of Freud and Burlingham [58], who found that children whose parents provided greater support and consistency in their daily lives in London during World War II had lower levels of psychological distress. The findings from the present study suggest that services that enhance parental as well as peer support may serve to protect immigrant youth from depression and/or PTSD. Services that offer support for families, such as social support groups for parents on communicating with children, may strengthen families in their capacity to provide a buffer for distress among youth. Peer-support programs in the schools, involving education on mental health, may help to reduce the social isolation often experienced by those suffering from depression and/or PTSD as well as identify youth who need referral for further assistance (e.g. youth experiencing substance use problems or suicidal thinking).

Results from this study also have implications for access to mental health services. It has been documented that immigrant youth underutilize mental health services [11], and despite a significant level of symptoms reflecting depression and PTSD in this population, only 1.8% of Haitian immigrant youth were seeing a mental health professional at the time of the present study. However, there is a need to identify what kinds of mental health care are appropriate for immigrant and refugee youth. Fazel and Stein [13] indicate that more empirical data on the effectiveness of therapeutic interventions among refugee children are needed. As suggested by Summerfield [59], perhaps 'working through' trauma may not be very helpful, however, mental health care that serves to enhance coping mechanisms (potentially through cognitive-behavioral methods) and strengthen social support networks may be more useful. Fox et al. [60] demonstrated a reduction in depressive symptoms among refugee children who participated in a school-based intervention that focused on skills-building and coping. Similarly, Kataoka et al. [47] observed an improvement in post-traumatic stress disorder and depressive symptoms among immigrant children in the U.S. who participated in a school-based cognitive-behavioral group therapy program. Eisenbruch [57] argues that the school can serve as an ecosystem for "preventive intervention" for refugee children, indicating that school-based programs are useful for primary prevention but may also serve as an excellent setting for secondary prevention, i.e. identifying children with psychological distress and making referrals for appropriate mental health treatment in the healthcare system.

There are a number of limitations in this study. The stratified sampling procedure was not accounted for in the analyses and findings on prevalence rates may be biased and represent overestimates. In addition, given that all youth were enrolled in school, there may also be an underestimate of the prevalence of depression and PTSD since students with higher levels of symptoms may be at risk of dropping out of school [61,62]. Another factor that can have an affect on the assessment of depression and PTSD includes consideration of the cross-cultural validity of the questionnaires. Although a formal validation study is beyond the scope of the present paper, bivariate and multivariate analyses support the construct validity of these assessments, since expected risk factors were associated with PTSD and depression. Another limitation is that assessments relied on self-report of youth. The BED assessments could have been augmented by clinical interviews, however, limited resources for the study prevented this. In addition, the data collection was completed for this study eight years ago. However, given the limited funding available during the past eight years for mental health programs, particularly for minority or immigrant youth, the number of agencies/programs targeting Haitian youth unfortunately has not changed significantly. Therefore, the findings from this study are still relevant for Haitian American youth, primarily among those enrolled in public schools in urban centers in the U.S. Finally, the study was cross-sectional in nature. Risk factors and symptoms were assessed at the same time; therefore, the results emphasize factors "associated" with depression and PTSD and the direction of these relationships cannot be ascertained. In this regard, the analysis was exploratory and does not allow for causal inferences.

## Conclusions

A significant level of depression and post-traumatic stress disorder was observed in the present study reflecting a need for enhanced services in the schools and improved access to mental health services for Haitian immigrant youth. Depression and PTSD in adolescence have the potential to result in poor psychosocial outcomes and can have long-term implications. The broader context, including the safety levels of U.S. cities where many new immigrants and refugees with a history of trauma have resettled, should be considered, given that it appears that a continued lack of security in urban environments can result in a persistence of PTSD symptoms among youth [6]. While it is acknowledged that youth can serve as a tremendous resource for one another and that refugee and immigrant youth have often demonstrated a great deal of resiliency, more resources are needed at the policy and program levels to ensure that high risk youth receive much-needed prevention services and mental health treatment to prevent negative outcomes, including the risk of suicide, as they transition into adulthood.

## Competing interests

The authors declare that they have no competing interests.

## Authors' contributions

MCSF was responsible for the study design, implementation, and served as primary author of the manuscript. TSB participated in drafting of the manuscript. LM was the author primarily responsible for study implementation and also participated in drafting of the manuscript. MK collaborated in development of study design and drafting of the manuscript. KM and ACM participated in study implementation and drafting of the manuscript. CM and JSM were also involved with drafting of the manuscript. All authors have read and approved the final manuscript.

## Pre-publication history

The pre-publication history for this paper can be accessed here:

http://www.biomedcentral.com/1471-2458/9/482/prepub
